# Structure-Activity Relationship of Cannabis Derived Compounds for the Treatment of Neuronal Activity-Related Diseases

**DOI:** 10.3390/molecules23071526

**Published:** 2018-06-25

**Authors:** Cristina Prandi, Marco Blangetti, Dvora Namdar, Hinanit Koltai

**Affiliations:** 1 Department of Chemistry, University of Turin, 10125 Torino, Italy; cristina.prandi@unito.it (C.P.); Marco.blangetti@unito.it (M.B.); 2 ARO, Volcani Center, Rishon LeZion 7505101, Israel; namdardv@gmail.com

**Keywords:** *Cannabis sativa*, structure-activity relationship, phytocannabinoids, endocannabinoids, cannabinoid receptors, neuronal diseases, Parkinson’s disease

## Abstract

*Cannabis sativa* active compounds are extensively studied for their therapeutic effects, beyond the well-known psychotropic activity. *C. Sativa* is used to treat different medical indications, such as multiple sclerosis, spasticity, epilepsy, ulcerative colitis and pain. Simultaneously, basic research is discovering new constituents of cannabis-derived compounds and their receptors capable of neuroprotection and neuronal activity modulation. The function of the various phytochemicals in different therapeutic processes is not fully understood, but their significant role is starting to emerge and be appreciated. In this review, we will consider the structure-activity relationship (SAR) of cannabinoid compounds able to bind to cannabinoid receptors and act as therapeutic agents in neuronal diseases, e.g., Parkinson’s disease.

## 1. Introduction

The marijuana plant, *Cannabis sativa* L., produces hundreds of secondary metabolites. *C. sativa* produces a diverse group of isoprenylated resorcinyl polyketides commonly named phytocannabinoids. The ‘Lego-like’ building pathways of phytocannabinoids, via the non-enzymatic transformations induced by heat, light, and atmospheric oxygen, result in different resorcinyl side-chain, or variation in oligomerization degree of the isoprenyl end, creating alkyl- and a β-aralklyl chemotypes [1]. These include around 110 characteristic phytocannabinoids [2,3], the most studied of which are dronabinol (Δ^9^-tetrahydrocannabinol, THC) and cannabidiol (CBD). This plant has been known for thousands of years for its effect on the human body [2,3]. The psychoactive effect of *C. sativa*, produced by only one of its hundreds of constituents [4,5] presumably led to its cultivation more than 6000 years ago [6,7,8]. Ancient *C. sativa* is believed to be used for various social and ritualistic purposes, and even in palliative and medicinal applications [9,10,11].

Today, *C. sativa* is used medicinally to treat various medical indications. The integrated inventory of these compounds and their biological macromolecular end-points highlight the opportunities that phytocannabinoids offer to access desirable drug-like effects, beyond the one associated with the narcotic target CB1 [1]. The main active compound with psychotropic effects produced by the plant THCA, heated into the decarboxylate active THC [4], is probably the compound that alleviates chronic neuropathic pain [12,13,14], nausea, headaches, and fatigue. However, other active compounds of *C. sativa* may account for their additional medical activities in various tissues and body parts, such as epilepsy [15], chemotherapy-induced nausea [16], anorexia [17], multiple sclerosis spasticity [18,19,20,21], fibromyalgia and rheumatoid arthritis [12,13,14], glaucoma intraocular pressures [22], and asthma-associated dyspnea [23]. 

Besides phytocannabinoids, *C. sativa* produces more than 400 other phytochemicals including terpenoids and terpenes, flavonoids, and hydrocarbons [24]. Although a complete and unified inventory of phytocannabinoids has recently been published [1], there remains much to learn about the activity of the different phytochemicals, their modes of action on human body and their structure-activity relationships (SAR). In this review, we will focus on the SAR of natural and synthetic compounds of *C. sativa* that may bind G-protein-coupled cannabinoid receptors and may be beneficial in the treatment of neuronal diseases, e.g., Parkinson’s disease. 

### 1.1. Cannabinoids Receptors and Endocannabinoids

Three types of G-protein-coupled cannabinoid receptors (GPCR) are known to-date—first, CB1, cloned in 1990 and CB2, cloned in 1993 [25,26] were widely recognized and studied as cannabinoids effective targets [27]. Much later, a third G-protein-coupled cannabinoid, receptor 55, was suggested as CB3 [28,29].‏ The CB3 shares several cannabinoid ligands with the two previously recognized GPCRs, but with only low homology to the classical cannabinoid receptors [30]. Its pathophysiology is still vague and its functions in the central nervous system are not yet understood, although CB3 was shown to be expressed in several brain areas [31]. 

Evidence also suggested that cannabinoids bind to and act via nuclear, peroxisome proliferator-activated receptors (PPARs, with three subtypes α, β (δ) and γ) [32]. Cannabinoid receptors are distributed in the human body, mainly in the central nervous system, but also in other peripheral tissues including the spleen, the reproductive, urinary and gastrointestinal tracts, the endocrine glands, the arteries and the heart [33]. The existence of additional cannabinoid receptor subtypes in the endocannabinoid system was investigated [34,35,36,37]. Modulation of the endocannabinoid system using *C. sativa* has promising therapeutic effects in the treatment of various disorders, such as neurodegenerative diseases [38], epilepsy [39], cognitive deficits [40], and others. However, producing cannabinoid-derived drugs to treat these disorders by regeneration or modification of the endocannabinoid system is highly challenging and has yet to be achieved [41,42]. 

Endocannabinoids are produced in the body when needed, under stress, or in response to synaptic activity [43]. The most studied endocannabinoids are the orthosteric anandamide (AEA) and 2-AG [44,45]. They are considered to be dominant and are agonists for CB1 and CB2 receptors, with higher affinity to CB1 binding [46]. Further pharmacological characterization is still needed to thoroughly understand the physiological roles of endocannabinoids and their modes of action [42]. Nevertheless, pharmacological manipulation of endocannabinoid levels may provide new opportunities to regulate the endocannabinoid system and treat the related disorders [47].

### 1.2. Phytocannabinoids

**Figure 1:**
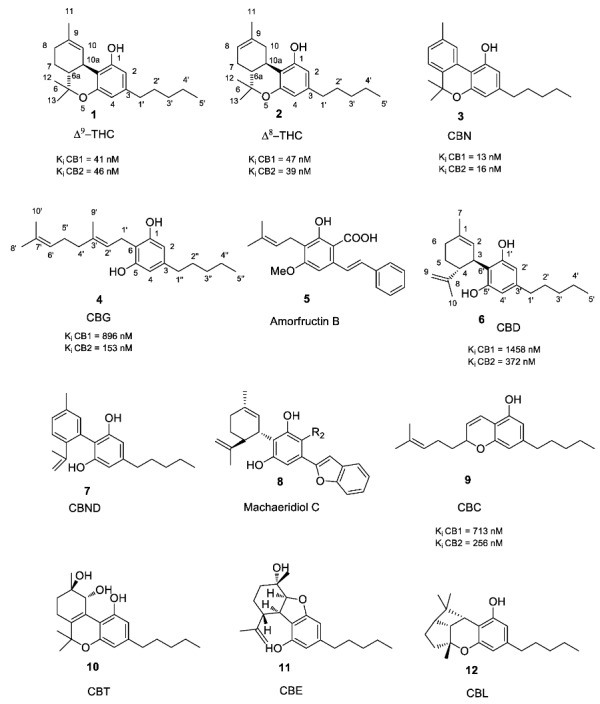
Structure of narcotic phytocannabinoids Δ^8^-THC, Δ^9^-THC, CBN with high affinity for ligands CB1 and CB2 and of non-narcotic phytocannabinoids CBG and CBD. Numbering system and binding affinities are reported.

Originally, the term “cannabinoid” referred to a homogeneous class of monoterpenoids typical of *C. sativa* L. More recently, the term “cannabinoids” has been extended to all those compounds showing an affinity for the GPCR known as cannabinoid receptors, CB1 and CB2, independently from their monoterpenoid skeleton (very little is known about GPR55, also named CB3, in this respect). The endogenously produced analogues showing affinity for CB1 and CB2 are known as endocannabinoids. To differentiate from this latter class of compounds, the term phytocannabinoids has been introduced to emphasize the botanical origin of these cannabinoids. Among the known plant-derived cannabinoids, the most abundant are tetrahydrocannabinols (THCs), cannabidiols (CBDs), and cannabinols (CBNs), followed by cannabigerols (CBGs), cannabichromenes (CBCs) and cannabinodiols (CBNDs, Figure 1) [48]. 

Classical phytocannabinoids are tricyclic terpenoid compounds bearing a benzopyran moiety soluble in lipids and non-polar organic solvents. The phenolic compounds are soluble as phenolate salts form under basic conditions. In the plant, the carboxylated form of the cannabinoids is more abundant, named “acid” and indicated as THCA, CBDA, CBGA and similar. The psychoactive compounds are the decarboxylated form of the varieties, i.e., THC, etc. There are some variations in the length of the C-3 side chain, pentyl being the most common but *n*-propyl derivatives are also well known. The *n*-propylated analogues with their shorter chain are named using the suffix “varin” and indicated as THCV, CBDV and similar [49]. 

As mentioned above, phytocannabinoids are classified, based on the resorcinol side-chain, into two main classes: (a) alkyl and (b) β-aralkyl (Table 1). As the β-aralkyl side chain derives from an aromatic starter, its residue replaces the alkyl group of the parent compounds. Subclasses of these two main classes are further identified based on the nature of the side-chain and on the presence of O-bridges with a resorcinol core [1]. 

#### 1.2.1. Cannabigerol (CBG) Compounds

Compounds belonging to this class do not show psychotrophic activity and are characterized by the presence of a linear non-oxygenated isoprenyl residue. Apart from the decarboxylated compounds CBG and CBGV (Figure 1) and their carboxylic parent forms (R_2_ = COOH, CBGA), all other CBGA related compounds are minor constituents in *cannabis* production. They show low affinity for CB1, even though prenylogation increases affinity for CB2. In addition, a quinone derivative of CBG possibly binds at the PPARγ receptor [50]. Indeed, as typical of CBG belonging to the β-aralkyl class, amorfructin B (**5**, Figure 1) [51,52] has been demonstrated to be a powerful ligand of PPARγ [53].

From the basic formula of CBG, the plant further converts and produces other cannabinoids, mainly CBC, CBD or THC, depending on the enzymes active in the process. Degradation of these compounds occurs spontaneously in the plant and results in CBN, among others. Here, we explore the biosynthesis pathway of these major cannabinoids. 

#### 1.2.2. Cannabichromene (CBC) Compounds

In this type of phytocannabinoid, the isoprenyl side chain is oxidatively fused to the resorcinol ring. The occurrence of CBC in many strains of *C. sativa* is often associated with Δ^9^-THC, suggesting a common biosynthetic path from the common precursor CBG. Natural CBC is racemic, shows a blue fluorescence under UV light, and does not exhibit any affinity for CB1 receptors [54]. 

#### 1.2.3. Cannabidiol (CBD) Compounds

The non-narcotic CBD is the major phytocannabinoid component in fiber hemp. The oxidase involved in the formation of the non-narcotic CBD from CBG are not related to those involved in the formation of CBC and THC. The independence of the biosynthetic pathways leading to CBD and to Δ^8^-THC and Δ^9^-THC, as well as the observation that the two compounds are not interconverted in cannabis tissues [55] increases the possibility of separating the narcotic effects from the therapeutic ones.

The elucidation of the absolute configuration was made based on the correlation with natural (-) menthol. For the compounds of the same class of the β-aralkyl series, only a few compounds have been isolated with absolute and/or relative configurations different from those isolated from cannabis. Machaeridiol A, B, C (**8**, Figure 1), used as antimalaric agents, are representatives of this class [56].

#### 1.2.4. Tetrahydrocannabinol (THC) Compounds

The most abundant and best-known component of this class is Δ^9^-THC, alongside a plethora of minor constituents that may result from degradation or modifications of Δ^9^-THC itself. In Δ^8^-THC, the double bond remains in a thermodynamically more stable position, and, as a result, a shift of the double bond from nine to eight positions is the preferred process [1]. The comprehensive stereochemistry of the structure of Δ^9^-THC was completely elucidated in 1964 [1]. Δ^9^-THC is less stable than its isomer Δ^8^-THC and easily undergoes isomerization of the double bond or to abstraction of the H in position 10a (both benzylic and allylic) and elimination to a conjugate diene, possibly a precursor of the aromatic CBN. The native form of Δ^9^-THC is represented by a mixture of two carboxylated pre-cannabinoids, Δ^9^-THCA and Δ^9^-THCB. The carboxylic derivative Δ^9^-THCA shows potent neuroprotective activity, which is worth considering for neurodegenerative and neuroinflammatory diseases [57]. Δ^9^-THC acts as a partial agonist of CB1 and CB2 [58]. 

#### 1.2.5. Cannabitriol (CBT) and Cannabelsoin (CBE) Compounds

These are minor constituents of cannabis extracts, occurring in only part of *C. sativa* sub-species. CBT (**10**, Figure 1) was the first to be recognized and isolated [59] and only a decade later its chemical structure was recognized [60]. CBE (**11**, Figure 1) is produced by the plant in negligible amounts but is also a mammalian metabolite of CBD [61].

#### 1.2.6. Cannabinoids Degradant: Cannabinol (CBN) and Cannabicyclol (CBL) Compounds

Cannabinol was the first phytocannabinoid structurally characterized. Cannabinol and its derivatives are now considered to be the oxidative by-product of the degradation process of THC and CBD compounds. CBC is photo-degraded into CBL. The cannabinoid degradants CBN and CBL (**12**, Figure 1) show low affinity for both CB1 and CB2. 

#### 1.2.7. Miscellaneous Cannabinoids

There are other cannabinoids not categorized in a class: 10-oxo-Δ-6a-tetrahydrocannabinol (OTHC); Cannabichromanon (CBCF); Cannabifuran (CBF) and dehydrocannabifuran (DCBF); Cannabiglendol; Cannabiripsol (CBR); Cannabicitran (CBT); Δ^9^-cis-tetrahydrocannabinol (cis-THC); and Tryhydroxy-Δ^9^-tetrahydrocannabinol (triOH-THC) [62]. However, their relative amounts in the plant are low.

## 2. Discussion 

### 2.1. Structure-Activity Relationship (SAR) of Cannabis-Derived Compounds for the Cannabinoid Receptors

The main goal in the study of the structure-activity relationship (SAR) of cannabis-derived compounds for the cannabinoid receptors is understanding the receptor binding sites. Currently, only the crystal structure of the human cannabinoid receptor CB1 has been fully achieved [63]. Deciphering the SAR of phytocannabinoids may help further understand the pharmacology and medicinal chemistry of the cannabinoid receptors in order to develop targeted remedies [64]. Moreover, understanding the SAR mechanisms of cannabinoids with their receptors may help the clinical research find new substances with therapeutic effects [65] and with minimized side-effects on cognitive functions.

**Figure 2:**
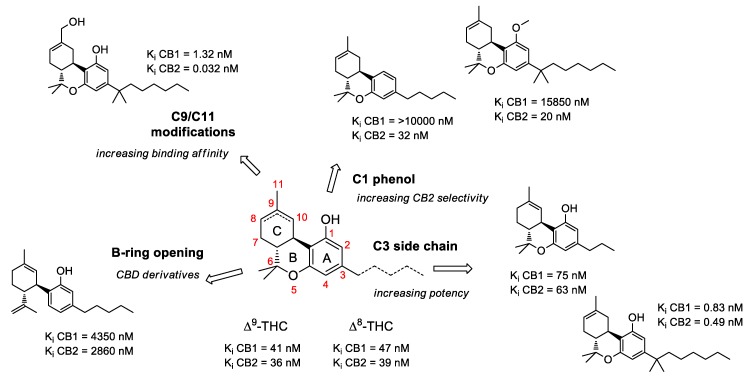
Selected examples of common chemical modifications on tetrahydrocannabinol skeleton.

Over the past 60 years, considerable research in medicinal chemistry has been carried out towards the SAR development of the natural classical cannabinoids; only in 1986 did the research group of R. K. Razdan analyze the SAR of about 300 cannabinoid analogues based on their activity in different animal models [66]. After the identification of Δ^9^-THC in 1964 [67], several chemical modifications of the side chain and/or the tricyclic scaffold led to the characterization of families of potent selective ligands that could be involved in the activation of the main cannabinoid receptor. It has been shown that the *n*-pentyl chain at the C-(3) position (Figure 1), incorporated during the biosynthesis of olivetolic acid [49], represents the key pharmacophoric group of THC [68,69] and modification in this side chain leads to critical changes in the affinity, selectivity and pharmaco-potency of these ligands relating to the cannabinoid receptors (Figure 2). 

In general, a shorter chained alkyl group reduces the potency of the compound to interact with the receptor. In THC, for example, a propyl group at C-(3) creates THCV (tetrahydrocannabivarine), which shows a 75% reduction in the potency to CB1 [66]. An increase in the number of carbon atoms (hexyl, heptyl, or octyl) leads to a respective increase in affinity and potency to interact with the cannabinoid receptors [70,71]. The length of the C-(3)-side chain of THC directly corresponds with CB1 and CB2 binding affinities, as an increase in chain length leads to an increase in binding affinity with the cannabinoid receptors [70]. Based on these ideas, various analogues of different carbon chains and rings, with or without heteroatom incorporation, may suggest the prediction of a SAR profile for a given structure, such as the THC scaffold. Besides the well-established study of the candidate target of SAR modulation based on the alkyl side chain, a number of other transformations in the tricyclic core of the cannabinoid structure have been carried out [72]. The cannabinoid compounds resulting from the pyran ring-opening reaction belong to the cannabidiol (CBD) derivatives, which demonstrate relatively low affinity to the CB1/CB2 cannabinoid receptors along with low psycho-activity [73]. Early SAR studies showed that the pyran ring in the cannabinoidic structure was not a requirement for cannabinergic activity in animal assays. However, several cannabidiol derivatives with high affinities for CB1 and CB2 receptors have been synthesized and thoroughly investigated [58]. Another possible structure modification on the THC scaffold is the C-(1) phenol group (Figure 1). THC analogues that lack the phenolic hydroxyl group altogether, or even those exhibiting minor modifications to their phenolic group, may demonstrate drastic changes in their pharmacological abilities. It was recognized that CBD derivatives that experienced etherification or elimination of the phenol group displayed significant selectivity for CB2. The C-(11) methyl group is another major pharmacophore where minor structural changes can significantly modulate receptor binding (Figure 2). Substitutions at this position do not confer selectivity when compared to analogues modified at the C-(1) phenol; however, the binding affinity may be greatly enhanced by this modification.

Δ^9^-THC is an agonist for both CB1 and CB2 receptors. Its analgesic properties [74] were often overlooked due to its psychotropic side effects resulting from its activation of the CB1 receptor. This has limited the clinical application of the cannabinoid dual agonists, despite the multiple potential benefits for the treatment of neurodegenerative diseases, among others [75]. Therefore, the potential of synthesized cannabinoid analogues that may exploit the therapeutic effects of cannabinoids without evoking the non-desired psychotropic properties is highly desired and extensive research in this direction is underway.

### 2.2. SAR of Cannabinoids and Their Receptors in Treating Neuronal Diseases

A possible prospect would be to study the SAR mechanism of synthesized cannabinoids, which do not evoke the non-desired side effects of phytocannabinoids. This may be achieved by the use of cannabinoids that would target the CB2 receptor only, as its activation does not lead to psychotropic side effects and makes it the ideal target for the treatment of several neurodegenerative diseases including Parkinson’s disease (PD) [76]. PD is a chronic disorder involving progressive degradation of the neuronal system and is the second most common neurodegenerative disease worldwide. No therapies are currently available to cure PD. The symptomatic therapies available today only improve patient quality of life [77]. In PD, dopamine neurons of the *substantia nigra* (i.e., “black matter”) of the brain degenerate leading to severe denervation of the striatum that affect motor activity. This irreversible damage leads to the typical motor symptoms observed in patients suffering from PD, including bradykinesia, rest tremor, and rigidity [77]. 

The cerebrospinal fluid of untreated PD patients was found to contain high levels of endocannabinoids [78]. Administering inhibitors of endocannabinoid degradation together with a D2 dopamine receptor subtype agonist reduced Parkinsonian motor deficits in in vivo models [79]. Furthermore, an increase in CB1 receptors was found in the nigro-striatal lesion of PD patients, and in models of nonhuman primates [80]. Several clinical studies and animal models suggest that antagonists to the CB1 receptor could have value in the treatment of levodopa-induced dyskinesia and PD symptoms, whereas agonists to CB1 receptor could prove useful in reducing levodopa-induced dyskinesia [81]. In addition, a quinone derivative of CBG has recently been shown to have neuroprotective activity against inflammation-driven neuronal damage in an in vivo model of PD by the possible involvement of different binding sites at the PPARγ receptor [50]. Indeed, it was demonstrated in a randomized, double-blind, placebo-controlled, crossover clinical trial that the nabilone, a cannabinoid receptor agonist, significantly reduces levodopa-induced dyskinesia in PD patients [82].

However, the CB2 receptor may also be important in PD treatment. CB2 was shown to be upregulated in glial elements in postmortem tissues of PD patients. Moreover, selective activation of CB2 receptors reduced pro-inflammatory mediators, confirming an inflammatory model and suggesting that CB2 may have an anti-inflammatory function in this disease [83].

Oral administration of *C. sativa* extract to stimulate CB1 receptor activity was reported to cause no improvement in PD symptoms [84], suggesting that cannabinoid activity may have to be verified, which may be accomplished in two ways. One would be to use a combination of compounds from *C. sativa* to improve activity. Note that the combination of *cannabis*-derived compounds is suspected to have a synergic effect [85]. Another option would be to synthesize cannabinoid analogues that may better bind cannabinoid receptors, preferably the CB2. Indeed, we acknowledge several efforts that have been made in the design of CB2 selective derivatives [86] and in the understanding of their structure-activity and structure−affinity relationships [87]. 

In this context, scientific research is focused on the development of molecular entities with high affinity for the cannabinoid receptor CB2. In recent years, a large number of CB2-selective synthetic compounds aimed at the treatment of neurodegenerative diseases have been developed around a wide variety of (hetero)aromatic scaffolds. A detailed literature survey about the development of synthetic CB2 ligands was recently extensively reviewed [88]. CB2 selective derivatives have been developed starting from mono- or bicyclic scaffolds bearing heteroatoms, bulky aliphatic or aromatic carboxamide groups, and either alkyl, aryl or arylalkyl substituents. Studies focused on the research of the molecular unit responsible for the affinity, the selectivity towards CB2 and the activity profile led to the design of a novel CB2 ligand for the treatment and early diagnosis of the neurodegenerative diseases. 

### 2.3. SAR and Activity Profiles of Several CB2 Selective Derivatives

**Figure 3:**
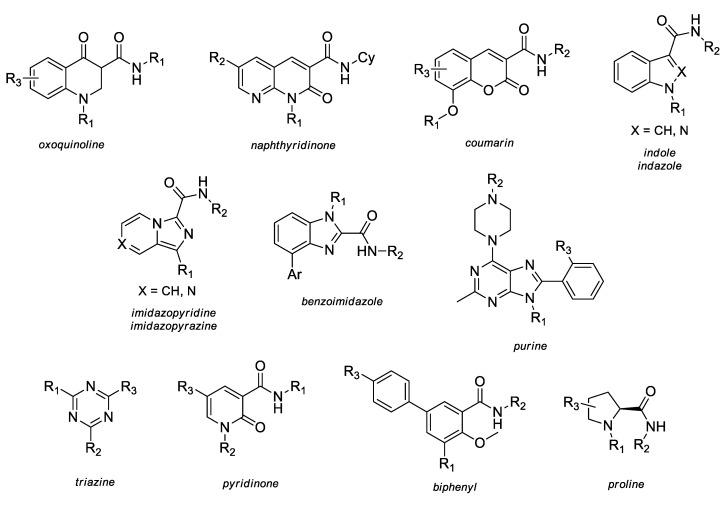
The (hetero)aromatic carboxamide scaffolds investigated in their SAR (structure-activity relationship) and activity profiles.

Several (hetero)aromatic carboxamide derivatives have been analyzed in terms of their SAR and activity profiles, including oxoquinoline; naphthyridinone; quinolinedione; alkyloxycoumarin; indole; indazole; imidazopyridine; imidazopyrazine; benzimidazole; purine; triazine; pyridinone; biphenyl; and proline (Figure 3). 

#### 2.3.1. Oxoquinoline 

Several substituents on the 4-oxoquinoline structure have been investigated [89,90,91,92]. High CB receptor affinities may be attributed mainly to the alkyl linear chains at C-(1) position with the *n*-pentyl group leading to the highest relative affinity to CB2. Bulky and lipophilic saturated-chain substituents of the 3-carboxamide functional group lead to high dual affinities. The highest affinity and selectivity for the CB2 receptor is achieved with an adamantyl ring. Substitution in the 6-position with aryl, alkyl, alkenyl, or alkynyl groups also lead to high selectivity for the CB2 receptor subtype [92].

#### 2.3.2. Naphthyridinone 

1,8-Naphthyridin-2(1*H*)-ones display high affinity for the CB2 receptor, which is strongly influenced by the *N*-(1) substituent, while the presence or the absence of an aryl substituent on the C-(6) confers a different activity profile [93,94,95].

#### 2.3.3. Coumarin 

A series of coumarin derivatives have been designed on the basis of a Comparative Molecular Field Analysis (CoMFA) model and developed by Han et al. [96]. The best CB2R agonist activity (EC_50_(CB2) = 0.144 μM, CB1/CB2 selectivity ratio = 69.4) was found for 8-butyloxy substituted compounds on R1. The incorporation of the 3-carboxamide *N*-atom in a piperidine ring decreased agonist potency, indicating that the presence of a tertiary amide function leads to the loss of agonist activity.

#### 2.3.4. Indole and Indazole 

SAR studies on indole derivatives revealed that among the *N*-indole carboxamide drugs the valinate and *tert*-leucinate methyl esters behave as potent agonists at CB1 and CB2 receptors. Recently, Longworth et al. [97] studied a series of 1-alkyl and 2-alkyl indazoles derivatives, where 1-alkyl isomers showed high CB1 agonist activity with nanomolar potencies (2.1−7.8 nM), where CB2 activity was less potent than CB1 activity. The 2-alkyl isomers displayed low potency towards both cannabinoid receptors.

#### 2.3.5. Imidazopyridine and Imidazopyrazine

These two series were tested towards both cannabinoid receptors and for their binding ability to plasma proteins. Imidazopyridines showed a higher agonist profile for the CB2 receptor than imidazopyrazine derivatives; moreover, the introduction of polar substituents in R2 increased the plasma protein binding ability. Overall, their potency for the CB2 receptor is modulated by modifications on the amide (R1) and amino (R2) functionalities [98].

#### 2.3.6. Benzimidazole

SAR studies on 2-arylmethyl or 2-aliphatic benzimidazole amides were performed [99]. Compounds bearing R1 = ethyl are more potent and more selective toward the CB2 receptor than *N*-unsubstituted derivatives. The derivative bearing a 2-chlorobenzyl substituent as R2 was the most potent compound toward the CB2 receptor, showing a 100-fold selectivity over the CB1 receptor. All the substitutions on the aryl in R2 led to an overall decrease of potency for the CB2 receptor and to the loss of CB1 receptor activity.

#### 2.3.7. Purine

Purinic ligands are potent CB2 agonists and show an excellent selectivity toward the CB1 receptor with good pharmacodynamic and pharmacokinetic profiles and water solubility [100].

#### 2.3.8. Triazine

Trisubstituted 1,3,5-triazines were identified as potent CB2 agonists by 3D ligand-based virtual screening. Several CB1 receptor antagonists or inverse agonists and CB2 agonists were developed, and the most potent derivatives of the series were identified in *N*-(adamantan-1-yl) substituted compounds with EC_50_ values up to 0.60 nM [101].

#### 2.3.9. Proline

Several proline derivatives were identified by a computer assisted drug design (CADD) approach based on a well-known series of CB2 receptor ligands by Hickey and co-workers [102]. Several (*S*)-isomers showed full CB2 agonist activities with high potencies (picomolar range) and a CB2/CB1 selectivity ratio higher than 750, while the corresponding (*R*)-isomers displayed a partial agonist profile with lower potencies toward the CB2 receptor. Several proline derivatives bearing different R1 and R2 substituents were also developed: hydroxyproline derivatives showed high CB2 potency and selectivity as well as the highly water-soluble δ-oxoproline derivatives; the latter demonstrated high metabolic stability.

#### 2.3.10. Pyridinone

Developed derivatives of 2-pyridinone showed high CB2 selectivity and affinity when *N*-substituted on the carboxamide with a large cycloalkyl ring, while substituent on the C-(5) was found pivotal for the activity profile [103]. 

#### 2.3.11. Biphenyl

SAR studies carried out on biphenylic carboxamides showed once more that a large cycloalkyl ring (cycloheptyl) on the carboxamide function improves affinity and selectivity for the CB2 receptor, while substituents in C-(5) and C-(4′) are mainly responsible for the activity profile [104,105]. 

**Figure 4:**
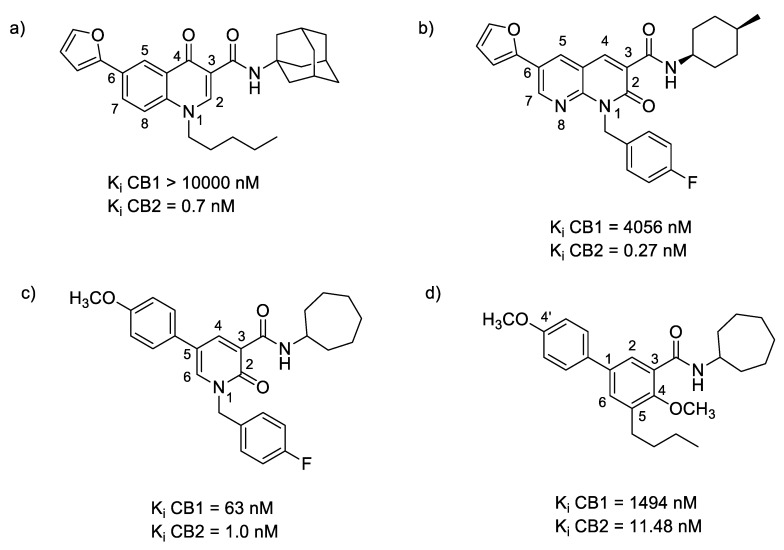
The (hetero)aromatic compounds which exhibit the highest affinity for CB2 receptors based on (**a**) oxoquinoline, (**b**) 1,8-naphthyridin-2(1*H*)-dione, (**c**) 2-pyridinone and (**d**) biphenyl structures.

In summary, oxoquinoline (Figure 4a), 1,8-naphthyridin-2(1*H*)-one (Figure 4b), 2-pyridinone (Figure 4c), and biphenyl (Figure 4d) scaffolds have shown the highest affinity toward a CB2 receptor subunit. An analysis of the common structural features of these compound classes reveals that high selectivity toward the CB2 receptor requires the presence of a carbonyl group and a carboxamide function linked to a cycloalkyl ring. Moreover, functionalization of the C-1 position and the presence of a nitrogen atom in the cycloalkyl ring improve the affinity, while the presence of a bicyclic ring is not mandatory to achieve good selectivity to CB2. Moreover, minor modifications of the cannabinoid structures has a dramatic effect on the pharmacological behavior of these compounds, switching the profile from being an agonist to inverse agonist or antagonist activity [88].

## 3. Conclusions

Studies of the mechanisms controlling neurodegenerative diseases, together with systematic observations of the cannabis treatments given to patients with neurodegenerative diseases, led to the recognition of the beneficial effect of cannabinoids on these illnesses. However, the very complicated assemblage of secondary metabolites in *C. sativa* extracts, together with the non-desired psycho-reaction accompanying cannabis use, led to the appreciation of the importance of the structure-activity relationship. SAR studies are essential to synthesize an appropriate cannabinoid with high therapeutic availability and low psycho-activity. The potency of CBD was marked as it has relatively low psychoactivity due to its low affinity to the CB1 receptor. In this paper, we show that different minor manipulations of the CBD structure may increase its affinity and binding ability to CB2. We also show that CB2 activation is involved in neurodegenerative conditions, such as PD, and thus activation of the CB2 receptor may be developed as a treatment for PD patients. The study of the SAR mechanisms between cannabinoids and their receptors, as well as the design of targeted cannabinoids-like compounds with higher SAR abilities, along with the understanding of CB2 enhanced reception role, put SAR studies at the cutting-edge in *C. sativa* studies and should be further investigated and clinically tested. 

## Figures and Tables

**Table 1:** Phytocannabinoids classification based on the nature of the resorcinyl side-chain.^1^

	Alkyl	βAralkyl
Cannabigerol (CBG)	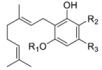	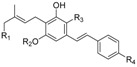
Cannabichromene (CBC)	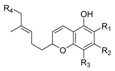	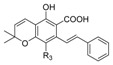
Cannabidiol (CBD)	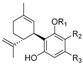	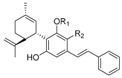
Tetrahydrocannabinol (THC)	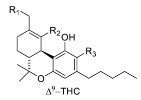	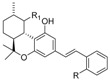
Cannabinol (CBN)	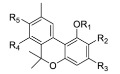	-

^1^ Only principal classes are reported and selected for the focus of this review. For a complete inventory, see [1].
